# Clinical profile of neonatal intestinal obstruction in northeastern Nigeria

**DOI:** 10.11604/pamj.2025.50.21.37554

**Published:** 2025-01-10

**Authors:** Samuel Wabada, Adewale Olaotan Oyinloye, Auwal Mohammed Abubakar, Rikin Uruku Christopher, Mohammed Sani Musa, Muhammad Habeeb Adamu

**Affiliations:** 1 Department of Surgery, Paediatric Surgery Unit, University of Maiduguri Teaching Hospital, Maiduguri, Borno State, Nigeria,; 2 Federal Medical Center Yola Adamawa State, Adamawa State, Nigeria

**Keywords:** Neonatal intestinal obstruction, clinical features, neonatal surgery, outcome

## Abstract

**Introduction:**

outcome of emergency neonatal surgery is still poor in developing countries due to a lack of trained workforce, cultural barriers, and poor understanding of the patient´s physiology. Aim: the focus of this study is to review the etiology and determine factors associated with poor outcomes of neonatal intestinal obstruction in northeastern Nigeria.

**Methods:**

a retrospective study of neonates aged 28 days at two tertiary Hospitals, the University of Maiduguri Teaching Hospital and Federal Medical Centre Yola was undertaken from February 2015-January 2020. Information on patients´ clinical features, duration of symptoms, diagnosis, treatment, and outcome were analyzed. Relationship between continuous and outcome variables was analyzed using ANOVA test, P-value < 0.05 is regarded as significant, confidence interval (CI) of 95%, while the correlation between categorical variables was analyzed with the Chi-square test.

**Results:**

the study had 147 (74.6%) boys and 50 (25.4%) girls (a boy-to-girl ratio of 2.9: 1). The mean weight of the cohort was 2.66±06 Kg. The most common causes of neonatal intestinal obstruction were anorectal malformations in 101 (51.3%), followed by intestinal atresias in 44 (22.3%), intestinal malrotations in 20 (10.2%), and Hirschsprung´s disease in 18 (9.1%). Most 124 (62.9%) neonates presented by the 5^th^ day of life were severely dehydrated and hypothermic with signs and symptoms of intestinal obstruction. Severe dehydration and hypothermia fever were associated with high post-operative mortality in these categories of neonates presenting late. Hence, neonates with prolonged duration of symptoms statistically correlate with high post-operative mortality P< .002, CI: 6.81-8.49

**Conclusion:**

anorectal malformations, intestinal atresia, and intestinal malrotation are common causes of neonatal intestinal obstruction in Nigeria.

## Introduction

Intestinal obstruction appears to be a common surgical emergency in neonates [1-4]. In the past sixteen years anorectal malformations, Hirschsprung´s diseases, intestinal atresia, and intestinal malrotations have been recognized as common causes of intestinal obstruction in neonates in Nigeria [5]. And, their post-operative outcomes have literally remained poor due to late presentation, lack of manpower, and poor facilities. This is in addition to unhealthy cultural practices, ignorance, and poverty that further derail the chance of survival of neonates with intestinal obstruction in our environment [6]. The study reviewed the morbidity and mortality associated with the clinical presentation of intestinal obstruction in neonates in two tertiary Teaching Hospitals in northeast Nigeria and determined factors that still affect their outcome despite facility upgrades and manpower development. Furthermore, the study also reviewed the variations in the etiology of intestinal obstruction in neonates from various regions in Nigeria whether they are due to the influence of regional cultural practices and habits.

## Methods

**Study design and site:** this was a two-center retrospective study of neonates aged ≤ 28 days who had an intestinal obstruction that was managed between February 2015 and January 2020 at the University of Maiduguri Teaching Hospital and Federal Medical Centre Yola. These two hospitals are major tertiary referral hospitals in northeastern Nigeria serving a population of around over 12 million 7% of the total population in the country.

**Study population:** all neonates with intestinal obstruction were admitted into the special baby care units of the University of Maiduguri Teaching Hospital and Federal Medical Center Yola between February 2015 and January 2020.

**Data collection:** data on patients’ demography, gestational age, birth weight, duration of symptoms, reasons for the delay in presentation, clinical features, diagnosis, associated anomalies, American Society of Anaesthesiology Score (ASA), surgery, and post-operative outcome were obtained from patients’ case files and operating registers.

**Selection of study participants:** only post-operative cases of congenital neonatal intestinal obstruction were considered for this review. Eighteen unoperated and two referred cases of post-operative adhesive intestinal obstruction were excluded from the study.

**Data analysis:** the data were analyzed using Statistical Package for the Social Sciences (SPSS) version 23 for Windows 10 (SPSS Armonk, NY: IBM Corp). The relationship between continuous variables and postoperative mortality was analyzed using ANOVA test, with P-value < 0.05 regarded as significant, and a 95% confidence interval (CI). Chi-square was used in the correlation of the relationship between clinical features, diagnosis, associated congenital anomalies, ASA score, treatment and postoperative outcome.

**Ethical approval:** ethical approval from the hospital ethical committees of the two hospitals. The approval numbers were OHRP-IRB00013572UMTH/REC/15/1027 and 11176FMCY/REC/15/0069 for University of Maiduguri Teaching Hospital, and Federal Medical Center Yola respectively.

## Results

**Demographic characteristics of the participants:** the records of 231 neonates aged ≤28 days were reviewed but only 197 records were available for substantial analysis. One hundred and twenty-seven (64.5%) records were from University of Maiduguri Teaching Hospital and 70(35.6%) from Federal Medical Centre Yola. There were 147 (74.6%) boys and 50 (25.4%) girls, (a boy; girl ratio of 2.9: 1). Mean admitting birth weight was 2.66±0.6 kg, with a range of 1.4-4.7 kg, and the mean gestational age was 38±.78 weeks, range 35-42 weeks. Mean duration of symptoms is 7.65± 5.95 range 1-28 days. Most 124 (62.9%) neonates presented by the fifth day of birth. Major reasons for late presentation in 80 (64.5%) of neonates were ignorance of the seriousness of the child´s condition, long distance to travel to the hospital, and lack of funds. No reason was given by the others.

**Clinical characteristics of participants:** regarding clinical features at presentation, 128 (65.0%) neonates had gross abdominal distension and bilious vomiting, out of which 113 (88.3%) had late onset of abdominal distention and vomiting due to large bowel obstructions, 64 (32.5) had associated constipation, while 5 (2.5) had associated jaundice and fever. Examination found 77 (39.1%) neonates were febrile and dehydrated, 57 (28.9%) dehydrated, febrile, and jaundiced, 52 (26.4%) dehydrated and hypothermic, and 11 (5.6%) were otherwise stable. The etiology of intestinal obstruction was anorectal malformations in 101 (51.3%) neonates, intestinal atresias in 44 (22.3%), and intestinal malrotations in 20 (10.2%) (Table 1, Table 2). Thirteen (6.6%) neonates had associated congenital anomalies. This included 9 (69.2%) Down´s syndromes in 5 (55.6%) with anorectal malformations, Hirschsprung´s disease, and duodenal atresias with 2 (22.2%) cases each. The rest of the associated congenital anomalies were hypospadias, atrial septal defect, congenital diaphragmatic hernia, and tracheoesophageal fistula.

**Table 1:** spectrum of aetiology of neonatal intestinal obstruction and mean duration of symptoms

Aetiology of intestinal obstruction	Mean duration of symptoms in days	N (%)
Anorectal malformation (ARM)	5.2±4.2, (range 1-27)	101(51.3)
Recto-urethral fistulae		70(35.5)
Recto-vestibular fistulae		9(4.6)
Perineal fistulae		10(5.1)
Covered anus		4(2.0)
Anal stenosis		3(1.5)
Anal agenesis without fistula		3(1.5)
Persistent Cloaca		2(1.0)
Intestinal atresia	10.86±6.4, (range 2-28)	44(22.3)
Ileal		38(19.3)
Type I		4(2.0)
Type II		14(7.1)
Type IIIa		15(7.6)
Type IIIb		2(1.0)
Type IV		3(1.5)
Jejunal		5(2.5)
Type I		1(0.5)
Type II		1(0.5)
Type IIIa		2(1.0)
Type IV		1(0.5)
Colonic		1(0.5)
Type III		1(0.5)
Intestinal malrotations	10.50±6.3, (range 1-22)	20(10.3)
Hirschsprung’s disease (HDx)	10.44±7.3, (range 2-27)	18(9.1)
Duodenal atresia (type I)	6.83±4.4, (range 2-18)	12(6.1)
Obstructed inguinoscrotal hernia	12.0±9.8, (range 2-19)	2(1.0)
Total	7.65±5.9, (range 1-28)	197(100.0)

**Table 2:** outlook of aetiology of neonatal intestinal obstruction in Nigeria

Aetiology	Our report	Ameh *et al*.	Adejuyigbe *et al*.	Ogundoyin *et al*.	Osifo *et al*.	Ekenze *et al*.	Olumide *et al*.
Anorectal malformation	101 (51.3)	104 (68.9)	73(49.4)	29 (22.3)	28 (39.4)	55 (43.0)	44 (16.0)
Intestinal Atresias	44 (22.3)	10 (6.7)	33(22.4)	2(1.5)	8(11.3)	20 (15.6)	67 (24.0)
Intestinal malrotations	20(10.3)	4 (2.6)	-	4 (3.1)	6 (8.5)	11 (8.6)	-
Hirschsprung’s disease	18(9.1)	11 (7.3)	19 (12.9)	18 (13.9)	8(11.3)	24 (18.7)	37 (13.3)
Duodenal atresia	12(6.1)	2(1.3)		2 (1.5)	-	10(7.8)	8 (2.7)
Obstructed inguinoscrotal hernia	2 (1.0)	11 (7.3)	8 (5.9)	5.3	4 (5.6)	8 (6.3)	15 (5.3)
Meconium ileus	-	1 (0.7)	-	-	-	-	-
Incarcerated exomphalos	-	5 (3.3)	-	-		-	-
Meconium plug syndrome	-	-	-	-	2 (2.8)	-	-
Neonatal sepsis	-	-	-	-	5 (7.0)	-	-
Necrotizing enterocolitis	-	-	-	-	4 (5.6)	-	-
Meconium peritonitis	-	-	-	-	3 (4.2)	-	
Pyloric stenosis	-	2 (1.3)	-	1(0.8)	-	-	75 (26.7)
Intestinal volvulus with congenital bands	-	1 (0.7)	14(9.4)	-	-	-	15 (5.3)

**Post-operative outcome of the study participants:** after resuscitation, colostomy for large bowel obstruction was performed in 105 (53.3%) neonates, followed by ileo-ileal anastomosis in 38 (19.3%) and Ladd´s procedure in 20 (10.2%). This suggests that colostomy was the most common surgical procedure in neonates in our environment (Table 3). The overall postoperative mortality was 32 (16.2%). More 23 (71.9%) of the mortalities occurred in neonates presenting late by the 5^th^ day as compared to 9 (28.1%) in those presenting early; this is statistically significant (p < 0.002. CI: 6.81 - 8.49). A statistically significant correlation was observed in patients´ clinical features and postoperative mortality, P<.000, χ^2^= 37.845. Neonates with a mean duration of symptoms of 13.0+/-6.9 days were more likely to die in the postoperative period as compared to those with a mean duration of symptoms lasting for 6.6+/-5.1 days (p < 0.002 95% CI: 6.61 - 8.49). The post-operative mortality was high among neonates presenting late that are febrile, severely dehydrated, and hypothermic (Table 4). Relationship between poor clinical features and high post-operative mortality was more in neonates that had ileal and colonic atresias, due to late-onset abdominal distension and vomiting presenting late with fever, jaundiced, severe dehydration, and a low ASA score.

**Table 3:** demography of neonatal surgical procedures and mortality

Surgical procedures	N (%)	Mortality N (%)
Sigmoid divided colostomy for anorectal malformation	87 (44.2)	5 (15.6)
Ileo-ileal anastomosis	38 (19.3)	13 (40.6)
Ladd’s procedure	20 (10.2)	4 (12.5)
Sigmoid colostomy+ extra mucosal biopsy for Hirschsprung’s disease	18 (9.1)	2 (6.3)
Anoplasty	14 (7.1)	
Duodenoduodenostomy	12 (6.1)	4 (12.5)
Jejunojejunostomy	5 (2.5)	3 (9.4)
Herniotomy	2 (1.0)	1 (3.1)
Total	197 (100.0)	32 (100.0)

**Table 4:** association of patients’ clinical profile, aetiology of intestinal obstruction and post-operative mortality

Variables	Survived N (%)	Died N (%)	χ^2^	df	P	N (%)
**Clinical features**						
**Symptoms**			37.845	**2**	0.00	
Abdominal distention (abd), distension, vomiting	122 (95.3)	6 (4.7)				128 (65.0)
Abdominal distention, distension, vomiting, constipation	41(64.1)	23 (35.9)				64 (32.5)
Abdominal distention, distension, vomiting, constipation, fever	2(40.0)	3(60.0)				5 (2.5)
**Signs**			37.385	**3**	0.00	
Febrile, dehydrated	74 (96.1)	3 (3.9)				77 (39.1)
Febrile, dehydrated, jaundice	50 (87.7)	7 (12.3)				57 (28.9)
Hypothermia, dehydration	30 (57.7)	22 (42.3)				52 (26.4)
Otherwise, stable	11 (100.0)	-				11(5.6)
**Aetiology of intestinal obstruction**			27.367	5	0.00	
Anorectal malformations	96 (95.0)	5 (4.9)				101(51.3)
Intestinal atresia	28 (63.6)	16(36.4)				44 (22.3)
Intestinal malrotations	16 (80.0)	4 (20.0)				20(10.2)
Hirschsprung’s disease	16 (88.9)	2 (11.1)				18 (9.1)
Duodenal atresia	8 (80.0)	4 (40.0)				12(6.1)
Obstructed inguinoscrotal hernia	1 (50.0)	1 (50.0)				2 (1.0)
**Associated congenital anomalies**			**4.237**	4	**0.375**	
Down’s syndrome	7(77.8)	2 (22.2)				9 (69.2)
Hypospadias	1(100.0)	-				1(7.7)
Aterial septal defect	1(100.0)	-				1 (7.7)
Congenital diaphragmatic hernia	1 (100.0)	-				1 (7.7)
Esophageal atresia-tracheoseophageal	-	1 (100.0)				1 (7.7)
**Treatments**			36.963	8	0.00	
Colostomy for anorectal malformation	82(94.3)	7 (5.7)				87 (44.2)
Anastomosis for ileal atresia	25 (65.8)	13 (34.2)				38 (19.3)
Ladd’s procedure for intestinal malrotations	16 (80.0)	4(20.0)				20 (10.2)
Anoplasty	15(100.0)	-				15(7.6)
Colostomy for hirschsprung’s disease (HDx)	1 (88.9)	2 (11.1)				12(6.1)
Duodenoduodenostomy	8 (66.7)	4 (33.3)				5 (2.5)
Jejunojenostomy	2 (40.0)	3 (60.0)				2 (1.0)
Herniotomy	1 (50.0)	(50.0)				
**Anaesthesiology score (ASA)**			57.047	**2**	0.00	
ASA II (mild systemic disease)	145 (94.2)	9 (5.8)				154 (78.2)
ASA III (severe systemic disease)	18 (45.0)	22 (55.0)				40 (20.3)
ASA IV (life threatening systemic disease)	2 (66.7)	1 (33.3)				3 (1.5)

**Figure 1:**
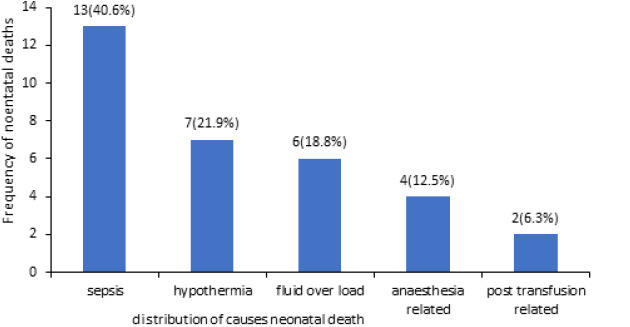
distribution of causes of death in neonatal intestinal obstruction

Due to these clinical features, 16 (13.4%) of neonates in this category died of post-operative sepsis and hypothermia. They already had diffuse peritonitis with gangrenous bowel segments at a presentation that resulted in their post-operative systemic organ failure despite attempts at careful antibiotic, fluid, electrolyte resuscitation therapy, and prevention of hypothermia. Hence, late presentation was the primary adverse outcome associated with high mortality due to sepsis and hypothermia in our environment (Figure 1). The secondary outcome variable was gestational age, neonates who had a mean gestational age of 36.3+/-1.11 weeks were more likely to die in the postoperative period as compared to those with a mean gestational age of 38.9+/-0.59 weeks (P<.000, 95% CI: 38.71-38.92). No significant association was observed between patients' birth weight and postoperative mortality rate (p > 0.258, 95% CI = 2.57 - 2.75).

## Discussion

This study and earlier ones showed high incidences of anorectal malformations as the cause of intestinal obstruction in Nigerian neonates [7-12]. Anorectal malformations were responsible for about 51.3% of cases of intestinal obstruction in neonates in this report. Regarding the epidemiology of etiology of intestinal obstruction in neonates in Nigeria, our report in the northeast had anorectal malformations, followed by intestinal atresias, intestinal malrotations, and Hirschsprung´s disease as the common causes of neonatal intestinal obstruction. Ademuyiwa *et al*. [13] in the southwestern part of the country had anorectal malformations, followed by Hirschsprung´s disease and malrotation of the intestine as the common causes of neonatal intestinal obstruction. Ameh *et al*. [14] in the northwest had anorectal malformations followed by Hirschsprung´s disease and intestinal atresias as the common causes of neonatal intestinal obstruction. This report and earlier ones agree that anorectal malformations were the common cause of neonatal intestinal obstruction. There were variable discrepancies in the epidemiology of other causes of intestinal obstruction in this report compared to earlier reports. These discrepancies could be sporadic as not enough evidence to suggest an environmental influence on the pattern of the epidemiology of etiology of neonatal intestinal obstruction between our report and earlier ones. But we believe high consanguinity, underage marriages, and consumption of herbal concoctions in pregnancy in our environment, which usually risk inheritance of genetic disorders and gastrointestinal insults probably accounted for the high incidences of anorectal malformations and intestinal atresias in this report.

The prevalence of duodenal atresia was low in this report similar to earlier reports [15,16]. This is probably due to early deaths from fluid and electrolyte losses in vomiting before reaching the hospital. Unlike, reports from Western countries where deaths from duodenal atresia are uncommon and so are said to be prevalent in such countries [17-19]. Similar to earlier reports in the region [20,21], late presentation is high among our neonates with intestinal obstruction. The average duration of symptoms at presentation was 7 days. The reasons for these neonates presenting late are ignorance about the serious nature of the child´s condition, poverty, and unhealthy cultural behaviors based on superstition, waiting to get the grandparents´ or the family elders´ approval before a child can be allowed to be sent to the hospital. Other reports across Nigeria have found similar reasons as major constraints for early presentation in children with intestinal obstruction [22]. The clinical profile of neonates with acute intestinal obstruction in our environment was poor due to late presentation, despite improved manpower and facility upgrades. A post-operative mortality rate of 71.9% was observed in neonates presenting late compared to the mortality rate of 28.1% in those presenting early; this was statistically significant (p < 0.002. CI 6.81 - 8.49). Furthermore, cofounding mortality was gestational age, neonates with a low gestational age of 36.3+/-1.11 weeks were more likely to die in the postoperative period as compared to those who had a gestational age of 38.9+/-0.59 weeks; this was also statistically significant (P<.000, 95% CI 38.71-38.92). Mortality in neonates with low gestational age was partly attributed to prematurity, overwhelming sepsis, and hypothermia.

Neonates presenting late often have severe dehydration, fever, jaundice, and hypothermia that delays early surgical intervention due to the time taken to correct sepsis, and fluid and electrolyte deficits. Thereby increasing the risk of bowel gangrene and perforations. Our report agrees with earlier reports [23], that emergency colostomies are the most frequently performed surgical procedure in neonates in Nigeria. This is because the most common cause of neonatal intestinal obstruction in Nigeria is anorectal malformations, which is a large bowel condition. This report put a post-operative neonatal mortality rate of 16.2%, which is an improvement on earlier reports of 30% [24]. We attributed the improvement to our experience, availability of more manpower, improved pediatric anesthetic expertise (and services), facility upgrade, and a better understanding of the physiology of the surgical newborn. Sepsis and hypothermia were responsible for 39.4% and 21.9% of our post-operative mortalities respectively (Figure 1), most of which occurred in neonates presenting late with hypothermia, severe dehydration, fever, and jaundice. Other studies have also found sepsis and hypothermia accounting for most of their post-operative deaths [25-27] mostly in neonates presenting late.

**Limitations:** this study could not provide data on the electrolyte profile of those neonates presenting late hence, no association could be established between electrolyte deficits and mortality. Furthermore, the influence of associated congenital anomalies and mortality was not considered in the study, especially amongst neonates presenting late. Associated anomalies are an important contributing factor to post-operative mortality in neonates in our environment.

## Conclusion

Anorectal malformations, intestinal atresias, intestinal malrotations and Hirschsprung’s disease are the common causes of intestinal obstruction in neonates in our environment. Mortality commonly follows post-operative sepsis and hypothermia in neonates presenting late with poor clinical features of intestinal obstruction. Prolonged duration of symptoms, poor clinical features and prematurity were independent variables that statistically predicted poor post-operative outcomes. Nevertheless, our study showed much improvement in the post-operative outcomes for the surgical treatment of neonatal intestinal obstruction compared to previous studies.

### What is known about this topic

- The outcome was poor due manpower shortages and lack of experience with neonatal surgery;
- Anorectal malformations and Hirschsprung’s disease are the common causes of neonatal intestinal obstruction in Nigeria;
- Late presentation is responsible for poor post-operative outcome of neonatal intestinal obstruction in Nigeria.

### What this study adds

- Post-operative mortality of neonatal intestinal obstruction has improved from what was known before-now is that we have more paediatric surgeons, improved paediatric anaesthesia, paediatric friendly equipment;
- Anorectal malformations and intestinal atresia are now the common causes of neonatal intestinal obstruction in Nigeria based on our study, probably because there are now more paediatric surgeons contribute to making the diagnosis and treatment of intestinal atresia;
- Socio cultural and economic barriers are responsible for late presentation in our study.
